# ASL-MRI-guided evaluation of multiple burr hole revascularization surgery in Moyamoya disease

**DOI:** 10.1007/s00701-023-05641-3

**Published:** 2023-06-16

**Authors:** Anders Lewén, Markus Fahlström, Ljubisa Borota, Elna-Marie Larsson, Johan Wikström, Per Enblad

**Affiliations:** 1 Department of Medical Sciences, Neurosurgery, Uppsala University, Uppsala University Hospital, SE 751 85 Uppsala, Sweden; 2 Department of Surgical Sciences, Neuroradiology, Uppsala University, Uppsala, Sweden

**Keywords:** Moyamoya disease, Moyamoya syndrome, Cerebrovascular reserve, Indirect revascularization, Multiple burr hole technique, Outcome

## Abstract

**Purpose:**

Moyamoya (MM) disease is characterized by progressive intracranial arterial stenosis. Patients commonly need revascularization surgery to optimize cerebral blood flow (CBF). Estimation of CBF and cerebrovascular reserve (CVR) is therefore necessary before and after surgery. However, assessment of CBF before and after indirect revascularization surgery with the multiple burr hole (MBH) technique in MM has not been studied extensively. In this study, we describe our initial experience using arterial spin labeling magnetic resonance perfusion imaging (ASL-MRI) for CBF and CVR assessment before and after indirect MBH revascularization surgery in MM patients.

**Methods:**

Eleven MM patients (initial age 6–50 years, 1 male/10 female) with 19 affected hemispheres were included. A total of 35 ASL-MRI examinations were performed using a 3D-pCASL acquisition before and after i.v. acetazolamide challenge (1000 mg in adults and 10 mg/kg in children). Twelve MBH procedures were performed in seven patients. The first follow-up ASL-MRI was performed 7–21 (mean 12) months after surgery.

**Results:**

Before surgery, CBF was 46 ± 16 (mean ± SD) ml/100 g/min and CVR after acetazolamide challenge was 38.5 ± 9.9 (mean ± SD)% in the most affected territory (middle cerebral artery). In cases in which surgery was not performed, CVR was 56 ± 12 (mean ± SD)% in affected hemispheres. After MBH surgery, there was a relative change in CVR compared to baseline (preop) of + 23.5 ± 23.3% (mean ± SD). There were no new ischemic events.

**Conclusion:**

Using ASL-MRI we followed changes in CBF and CVR in patients with MM. The technique was encouraging for assessments before and after revascularization surgery.

**Supplementary Information:**

The online version contains supplementary material available at 10.1007/s00701-023-05641-3.

## Introduction

Moyamoya angiopathy (MMA) is a progressive steno-occlusive vascular disease of the terminal internal carotid arteries followed by the development of typical collateral arterial network. Two clinical and pathologic entities are characterized by these morphologic changes: idiopathic moyamoya disease (MMD) and moyamoya “syndrome” (MMS). MMS is associated with a large group of heterogeneous conditions, e.g., certain hematological disorders, congenital anomalies, metabolic disorders, autoimmune diseases, neoplasms, and infectious diseases. MMA leads to disturbance of cerebral blood flow (CBF) and increased risk of ischemic or hemorrhagic stroke [18]. Patients at risk need to undergo revascularization surgery. Several neurosurgical techniques aim to improve CBF either by direct bypass or by various indirect revascularization methods or by combinations thereof [25]. Direct bypass offers immediate improvement of CBF but carries the risk of hyperperfusion syndrome [16] and hemorrhage [14]. Even though severe complications have also been described after indirect revascularization [15], the risks are probably lower with this technique, but the neovascularization takes time. The long-term outcome in both adults and children after indirect revascularization is comparable to or even better than direct methods [26]. Furthermore, indirect revascularization may provide blood flow to more than one vascular territory. The multiple burr hole (MBH) technique of indirect revascularization has been used in both adults and children [2, 8, 22, 23, 27, 35].

A challenge concerns patients with very mild or no symptoms regarding whether they should undergo revascularization surgery or be followed. Several aspects should be considered, such as symptoms, grading of MMA changes (e.g., from digital subtraction angiography (DSA)), presence and size of infarcts/ischemic lesions, assessment of CBF and the cerebrovascular reserve (CVR) after acetazolamide (ACZ) challenge [37]. The gold standard for CBF assessment is ^15^O-water positron emission tomography (PET), a method with disadvantages such as exposing the patient to radiation, need to use a cyclotron and arterial blood sampling. Arterial spin labeling (ASL) magnetic resonance imaging (MRI) [1, 13, 17, 20, 28, 34] has several advantages for evaluation of CBF in this patient group, especially considering the need for repeated examinations over several years, often in young MM patients. ASL-MRI has a good correlation with [^15^O]-water PET [12] and single-photon emission computed tomography [28] in MMA. Recently, ASL perfusion was used to study CBF after encephaloduroarteriosynangiosis [19] and bypass procedure [5, 33, 42]. However, evaluation of CVR using ASL-MRI has not been done after indirect revascularization using the MBH technique in MM patients. In this observational study, we assessed longitudinal changes in ASL, both CBF and CVR after acetazolamide challenge, in patients with MMA with and without MBH revascularization surgery.

## Patients and methods

Eleven patients (10 female/1 male, age 6–50 years) with MMD admitted to the Department of Neurosurgery at the Uppsala University Hospital, Sweden, 2015–2022 were eligible for this study.

### Assessment protocol

The patients had usually been examined with CT angiography or conventional MRI at a local hospital prior to admittance to our department. They were subsequently enrolled in our MMD protocol accordingly. Examinations were performed on a Philips Achieva 3.0 Tesla (Philips Healthcare, Best, the Netherlands) using a 32-channel head coil. A commercially available background suppressed 3D pseudo-continuous ASL (pCASL) gradient spin-echo read-out was acquired with a label duration of 1800 ms and post-label delay of 2500 ms to suppress potential artifacts due to delayed arterial blood flow. Repetition time was 4735 ms, echo time 10.7 ms, spatial resolution 3 × 3 × 6 mm^3^ and total acquisition time 5 min and 31 s. The labeling plane was placed perpendicular to the brain feeding arteries with the aid of a phase-contrast MR angiography survey, and no flow-crushing gradients were applied. CBF maps were calculated automatically by the scanner according to the model recommended by Alsop et al. [1]. CBF maps were acquired before intravenous injection of ACZ (1 g in adults or 10 mg/kg in children) (baseline) and repeated 5, 15, and 25 min after injection. Dynamic susceptibility contrast (DSC) perfusion using gradient echo-based echo planar imaging was acquired 30 min after ACZ injection. Repetition time was 1392 ms, echo time 29 ms, and spatial resolution 1.72 × 1.72 × 5 mm^3^. Gadolinium contrast (0.1 mmol/kg) was given at a 5 mL/s injection rate. DSC data were analyzed in NordicICE (NordicNeuroLabs, Bergen, Norway), and included motion correction. Parametric maps of time-to-peak (TTP) were generated. In addition, a structural 3D T2-weighted fluid attenuated inversion recovery (FLAIR) image, 3D contrast-enhanced T1-weighted (CE-T1WI) image and 3D time-of-flight MR angiography were acquired [9, 10].

CBF and TTP maps together with FLAIR images were registered to each patient’s CE-T1WI image. Left and right middle cerebral artery (MCA) territories were delineated by applying the inverse transformation from the vascular MNI template space by spatial normalization of a CE-T1WI image for each patient [9, 10, 30]. Cerebrovascular reserve capacity (CVR) was calculated as CBF after ACZ injection relative to CBF at examination baseline (see below). All the above mentioned processing steps were performed using the SPM12 toolbox (Wellcome Trust Centre for Neuroimaging, London, UK) [9, 10, 30]. In addition to ASL-MRI, most patients were also examined with DSA.

**Fig. 1:**
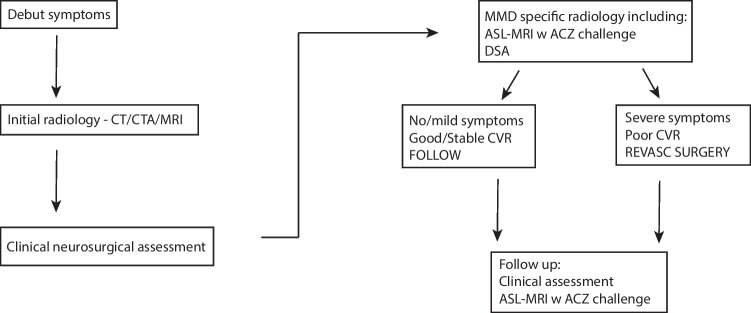
Schematic drawing of the MMA assessment protocol

Based on radiological findings and symptoms, patients were either followed with repeated ASL-MRI or planned for revascularization surgery. Criteria for surgery were severe symptoms with repeated ischemic attacks or progressive ischemic symptoms, ischemic lesions on MR, or decreasing and/or low CVR, i.e., either a clear decreasing trend on repeated ASLs and/or CVR around < 30% [10, 40]. Patients with very mild or no symptoms and CVR stable > 30% were monitored (followed). The MMD protocol is outlined in Fig. 1.

### Operative procedure

**Fig. 2:**
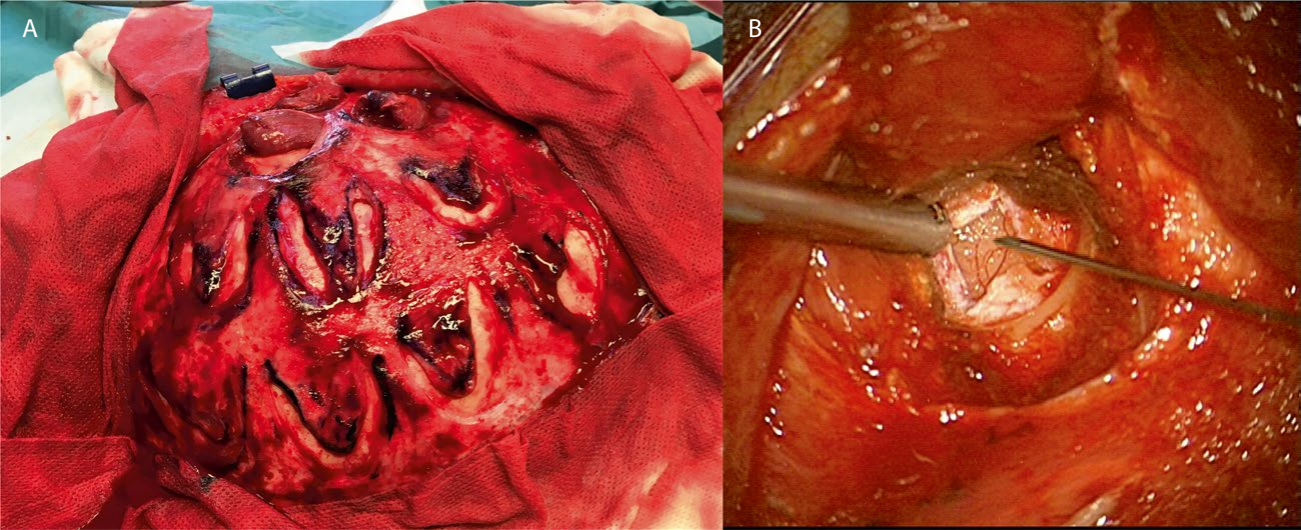
**A** Photograph from multiple burr hole surgery showing periosteal flaps. **B** After dural incision, the arachnoid, pia, and cortical surfaces were punctured with a needle. Thereafter, the periosteal flaps were laid on the brain surface and glued with tissue glue

The operative procedure was adapted from the techniques described by Sainte-Rose et al. [35] and later by Lavrysen and Menovsky [24]. All patients were kept on aspirin prophylaxis prior to and during surgery. Patients with bilateral changes were operated on each side on separate occasions; therefore, we did a standard T-shaped incision running in the midline and extending downward to the front of the ear on each side. After subgaleal dissection, the placement of 10–12 burr holes was marked out depending on the most affected region, starting approximately 4 cm from the midline and then in rows downward (Fig. 2). Usually, about three burr holes were placed under the temporal muscle. We carefully planned the locations of the burr holes so that the periosteal flaps would not compromise each other’s blood supply (Fig. 2). After periosteal flap incision, we used a standard 14/11 mm drill bone perforator followed by sharp opening of the dura. The arachnoid and pia mater were incised with a needle (Fig. 2). The periosteal flap was inserted and placed onto the brain surface and fixed with tissue glue (Tisseel®). If possible, we included some muscle with the periosteal flap in the temporal burr holes. A suction drain was used, and the wound was closed. Post-operatively, the patients were well hydrated, and systolic blood pressure was kept at the patients’ normal levels. When patients were fully mobilized in the post-operative ward, they were transferred to a normal ward, usually the following day.

### Follow-up

Post-operative clinical follow-up took place 4–6 weeks after surgery, and MR CBF imaging with ACZ challenge was performed at least 7 months postop and then approximately yearly (up to 48 months). Patients deemed not to need surgery were followed (monitored) with clinical examinations and evaluation of hemodynamic reserve every year.

### Statistical analysis

CVRC values were calculated based on regional CBF, using the following equation:

$$\text{CVR}\left(\%\right)=\frac{{\text{CBF}}_{\text{post-ACZ}}-{\text{CBF}}_{\text{baseline}}}{{\text{CBF}}_{\text{baseline}}} \times 100$$

for all post-ACZ injection examinations acquired (at 5, 15, and 25 min). The best response was used for further analysis. Values for the three major vascular territories (anterior cerebral artery, MCA, and posterior cerebral artery) were calculated, but only the MCA data are presented. For patients undergoing surgery, the CBF and CVR data obtained most recently prior to surgery were set as “baseline”, and the mean response in change in CBF and CVR was calculated on the basis of all of the patients’ postoperative examinations and related to this baseline as “Relative mean change from baseline in %”. Examinations were grouped into crude time intervals: postop 7–15 months, 22–28 months, and 34–48 months. Continuous data for CBF (ml/100 g/min) and CVR (%) were presented as means ± SD and medians with minimum and maximum ranges. All descriptive statistics were performed using the GraphPad Prism software (Dotmatics, Boston, MA, USA). No further statistical testing was done due to small sample size and heterogeneity of the material.

## Results

Patient characteristics are outlined in Table 1. In brief, the median age was 25.5 years, and all cases but one were female. Symptoms ranged from headache to transitory ischemic attacks cerebral infarcts and subarachnoid hemorrhage. Eight cases had classical bilateral MMD and three cases had unilateral findings (in this paper classified as MMS). One of the patients with unilateral changes had neurofibromatosis type 1 (NF1); in the other two patients, the genesis of the unilateral MMA changes was unknown. The median Suzuki stage was 3 (ranging from 2 to 4). In seven patients, surgery was performed using the MBH technique (12 hemispheres), and four patients were monitored without surgery (Table 1).

**Table 1:** Patient characteristics

Patient ID	Age/gender	Presentation	Etiology	Side	Suzuki stage	Treatment
1	14/M	TIA right side	MMS	Left	2	MBH left
2	25/F	Routine ex	MMS (NF1)	Right	4	MBH right
3	29/F	Paresthesia/headache	MMD	Bilateral	2–3	MBH right MBH left
4	46/F	TIA	MMD	Bilateral	4	MBH right MBH left
5	6/F	TIA right side + dysphasia	MMD	Bilateral	2–3	MBH left MBH right
6	26/F	Paresthesia/headache	MMD	Bilateral	3–4	MBH left MBH right
7	14/F	MCA infarct	MMD	Bilateral	4	MBH left MBH right
8	33/F	Facial pain	MMS	Right	2	Monitored
9	22/F	Previous surgery	MMD	Bilateral	_	Monitored
10	50/F	SAH	MMD	Bilateral	3	Monitored
11	16/F	Old infarct	MMD	Bilateral	4	Monitored

Age at surgery or diagnosis, M — male, F — female, TIA — transient ischemic attack, SAH — subarachnoid hemorrhage, MMD — moyamoya disease, MMS — moyamoya syndrome, MBH — multiple burr holes

### Overall results

**Fig. 3:**
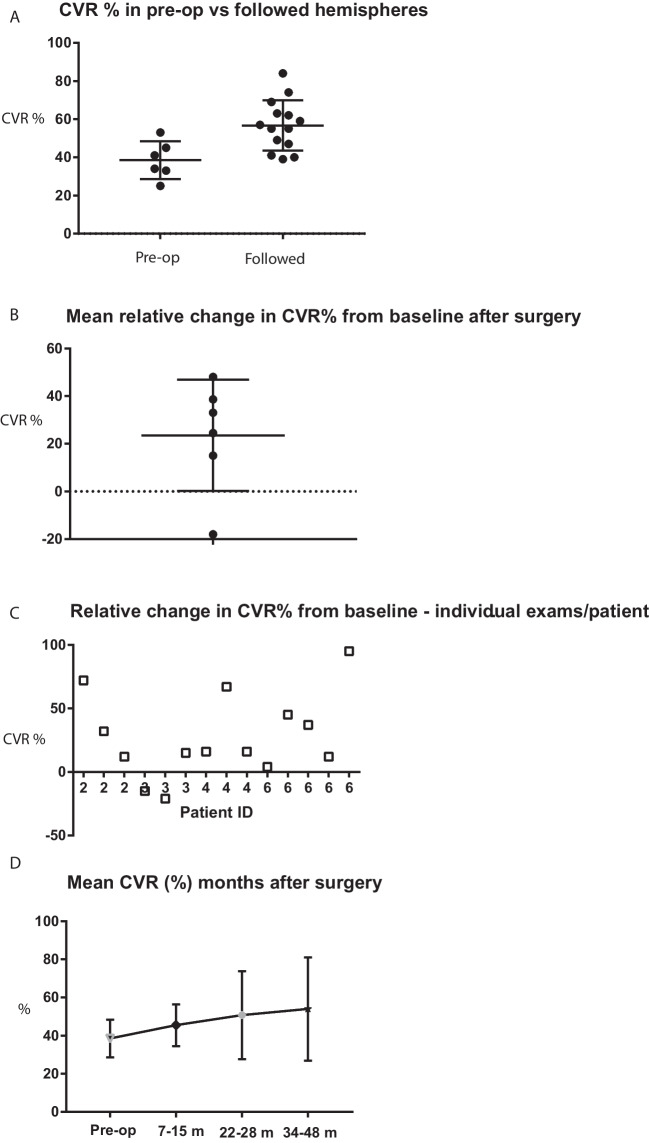
**A** Graph illustrating the mean pre-op CVR of 38.5 ± 9.9 (mean ± SD) in the middle cerebral artery territory in cases undergoing revascularization surgery with the multiple burr hole technique. Cases we decided to monitor but not operate (followed cases) generally had a higher CVR 56 ± 12%. **B** and **C** Comparing the relation between CVR pre- and post-op, the post-op CVR was in general higher compared to baseline. Data from six hemispheres in four patients are included (See Table 4 for details). **C** Results of relative change of CVR in individual patients for all examinations. These values were then used for mean calculations in each patient presented in Fig. 3B. **D** Over time, the increase in CVR was maintained after surgery but variability increased. Due to the inhomogeneity of the patients and examinations, no statistical testing between groups was done

Overall, in operated patients, the mean CBF in the affected MCA territory was calculated to be 46 ± 16 (mean ± SD) ml/100 g/min pre-op and 41.1 ± 12 (mean ± SD) ml/100 g/min at the first post-op ASL examination 7–12 months after surgery (Table 2). In the monitored patients, the mean CBF was 42 ± 12 (mean ± SD) ml/100 g/min. The mean of CVR prior to surgery in all operated cases (i.e., hemispheres) was 38.5 ± 9.9% (mean ± SD) in the affected middle cerebral artery territory (Table 3) vs. 56 ± 12 (mean ± SD)% in the monitored patients (Fig. 3A). The individual preoperative CVR response varied between patients (Table 4); the highest value was observed in patient #1, where the pre-op CVR was 60%, and the lowest pre-op CVR was observed in patient #2 where it was 25%. The actual post-op CVR ranged between 28 and 82% in individual patients (Table 4). In un-operated monitored cases, there were also large variations in CVR, between 39 and 84% (Table 4).

**Table 2:** Summary of estimated CBF (ml/100 g/min) in right (R) and left (L) hemisphere

CBF (ml/100 g/min)	Pre-op R	Post-op R	Pre-op L	Post-op L	Pre-op R + L	Post-op R + L	Relative mean change from baseline (%) R + L
Number of examinations	3	7	3	7	6	14	
Mean	43.3	40.4	48.7	41.7	46	41.1	− 5.3
SD	16	11.9	18.2	13.9	15.6	12.4	10.8
Median	42	40	56	43	49	41.5	− 3.8
Min	28	23	28	26	28	23	− 23
Max	60	55	62	60	62	60	5

*Pre-op* values reflect CBF prior surgery in affected hemisphere. R + L denotes combined results from both hemispheres. Relative mean changes were calculated from means of each patient’s postoperative results

**Table 3:** Summary of estimated CVR (%) in right (R) and left (L) hemisphere before and after surgery

CVR %	Pre-op R	Post-op R	Pre-op L	Post-op L	Pre-op R + L	Post-op R + L	Relative mean change from baseline (%) R + L
Number of examinations	3	7	3	7	6	14	
Mean	37	42	40	57	38.5	49.4	23.5
SD	14.4	19.8	5.6	15.1	9.9	18.6	23.3
Median	33	33	41	52	37.5	48.5	28.7
Min	25	26	34	39	25	26	− 18
Max	53	80	45	80	53	80	48

R + L denotes combined results from both hemispheres. Relative mean changes were calculated from the means of each patient’s postoperative results

**Table 4:** Individual CVR (%) values in each patient, data from all examinations. Patients denoted with * were not included in analysis of relative mean changes due to technical problems or lack of pre- or post-operative examinations

Patient ID	CVR% in affected MCA territory			
	*Pre-op (R)*	*Pre-op (L)*	*Post-op(R)*	* Post-op (L)*
1*		60		38, 62, 49
2	25		43, 33, 28	
3	33	34	28, 26	39
4**	45		52, 75, 52	
5*	–		19, 17, 25, 25	4, 10, 14, 30
6	53	41	55, 75	56, 45, 82
7*	25	33	–	
	*Not-operated*			
8	74, 84, 57, 59			
9	69, 55	49, 55,		
10	62, 41	63, 47		
11	39	40		

**Patient with bilateral MMD—CVR is presented only from right side since she was operated on the left side prior first CVR examination

In patients with both pre- and post-op examinations (*n* = 4), we compared the patients’ postoperative examinations (all dates) to patient baseline values (i.e., last CVR prior to surgery), and the mean relative change in CVR after surgery was + 23.5 ± 23.3% (mean ± SD) (Table 3 and Fig. 3B). One patient (#3) showed slightly lower CVR (relative change to baseline of 15 and 21% at 12 and 24 months postoperatively) on the right side (Fig. 3C). The other patients (*n* = 3) showed a positive relative change in CVR ranging from 4 to 95% postoperatively (Fig. 3C). Studying the CVR over time after MBH surgery, we noticed a sustained positive relative change, but with an increasing variability (Table 5 and Fig. 3D). For details regarding individual patients, see Supplement Fig. 1.

**Table 5:** Changes in CVR (%) at different time intervals after surgery

CVR (%)	Pre-op	7–15 months	22–28 months	34–48 months
Number of examinations	6	6	5	3
Mean	38.5	45.5	50.8	54
SD	9.9	10.9	23.1	27.1
Median	37.5	47.5	45	52
Min	25	28	26	28
Max	53	56	75	82

A short description of some illustrative patients included in the study is given below.

### Examples of cases revascularized by MBH surgery

#### Patient #2

**Fig. 4:**
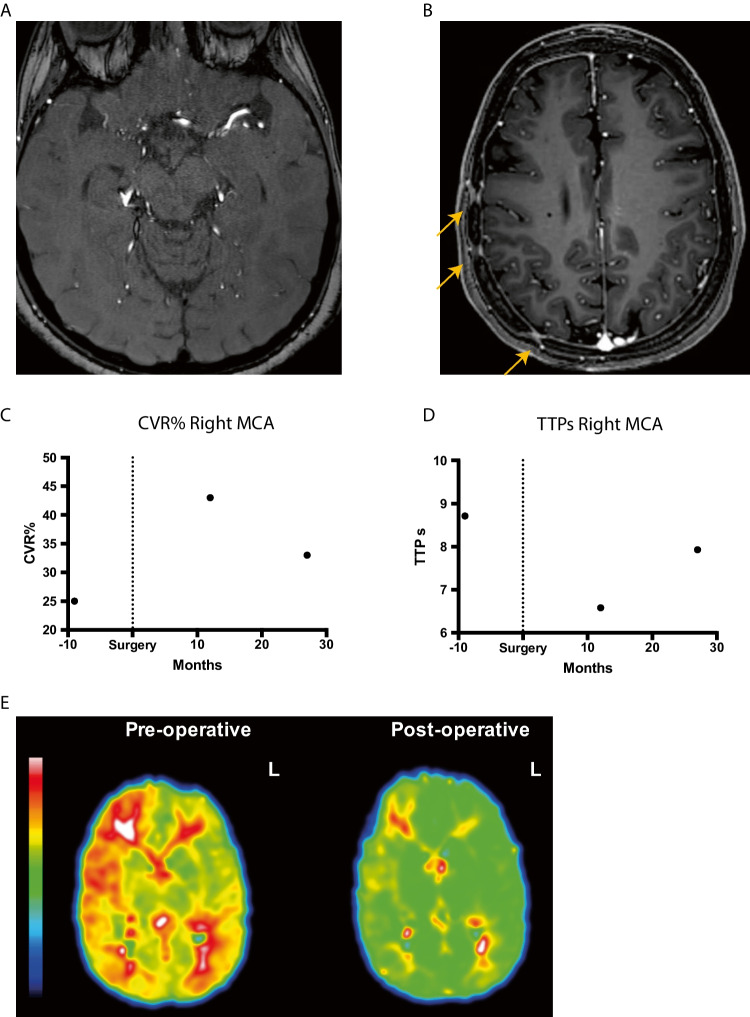
Patient 2 with right-sided MMS changes due to NF1. **A** Pre-op MRA showing occlusion of the right middle cerebral artery. **B** Post-op MRI showing contrast enhancement in multiple burr holes indicating ingrowth of blood vessels after MBH surgery. **C** Pre-op CVR was 25%, and 1 year later, it had increased to 43%. Second follow-up at 27 months showed a CVR decline to 33%, but no new symptoms. **D** and **E** Pre-op time-to-peak pre-op was similarly increased to 8.71 s, followed by a decrease to 6.58 s 12 months after surgery

A woman with neurofibromatosis type 1 (NF-1) treated with chemotherapy and radiation at < 6 years of age due to left-sided optic glioma. At age 16, a tumor appeared in the right basal ganglia. A stereotactic biopsy showed pilocytic astrocytoma, and she was treated with gamma knife irradiation at age 18. MRI at this point also showed occlusion of the right ICA and, to lesser degree, of the left M1 segment (Fig. 4). There were no symptoms, and changes were monitored for several years. At age 25, an ASL-MRI with ACZ challenge showed a lower MCA CBF on the occluded right side and a MCA CVR of 25% compared to 43% on the left side. TTP was 8.71 s on the affected right side and 7.59 s on the unaffected side. DSA showed an atypical right-sided MMS with Suzuki grade IV and also a small left-sided ophthalmic aneurysm. A right-sided MBH operation was performed at age 25. A 12-month follow-up, ASL-MRI with ACZ challenge showed that the right-sided CVR had improved to 43% (Fig. 4) and the left-sided CVR to 45%. In addition, contrast-enhanced signs of ingrowth of vessels in burr holes were seen. The post-op TTP was 6.58 s (right) (Fig. 4) and 6.1 s (left). At the latest follow-up at 48 months after surgery, the CVR had decreased somewhat to 28%, and future follow-ups are planned. No focal neurological symptoms have appeared after surgery.

#### Patient #5

**Fig. 5:**
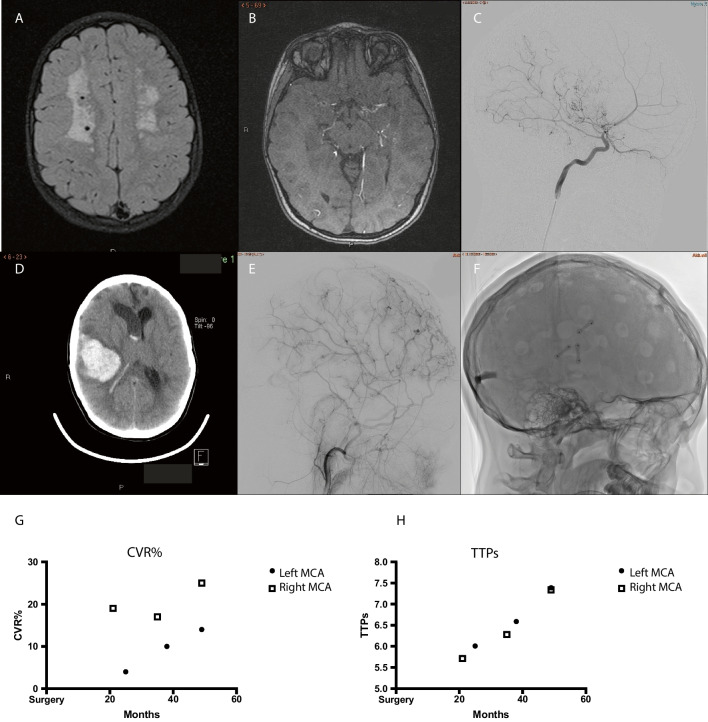
Patient 5 with bilateral MMD presenting at age 4. **A** Initial MRI showing chronic ischemia in white matter. **B** Follow-up MRI with time of flight sequence shows bilateral MMD, confirmed later with **C** DSA. At age 9, a revascularization procedure with multiple burr holes was performed. Three years after surgery, she developed an intracerebral hemorrhage on the right side **D** that was evacuated. A DSA was performed showing no aneurysms/AF fistulas. **E** Ingrowth of numerous blood vessels through the burr holes and improved perfusion were seen on DSA. **F** The location of the burr holes. **G** CVR was not established pre-op due to patient age. However, post-op we have been able to follow her CVR, which has generally been low, on the right side about 20% and on the left side between 4–14%. **H** TTPs has been low but has increased with time post-op

At debut, this 4-year-old girl presented with transient weakness in the right arm. Initial MRI gave suspicion of leukodystrophy (Fig. 5), but a follow-up MR after 12 months was more indicative of chronic ischemia with already some frontal atrophy. DSA showed typical bilateral MMD with a Suzuki grade of II–III (Fig. 5C). She was put on aspirin, and at age 9, she was operated with the MBH-technique bilaterally at 3-month intervals, starting on the left side. Since lengthy MR is not well tolerated in awake young children and we wanted to avoid general anesthesia, CVR was not examined before surgery. However, after surgery, 4 post-op MRI-ASL with ACZ challenge were done with the patient awake. CVR was 4, 10, 14, and 12% on the left side and 19, 17, 25, and 20% on the right side (Fig. 5G). Numerous ingrowths of blood vessels through the burr holes and reduction of chronic ischemia in the white matter were seen on MRI (not shown). Three years after surgery she developed a large intracerebral hemorrhage (Fig. 5D) in the right frontal lobe that needed emergency evacuation through a minimalistic approach. There were no apparent sequelae. DSA was performed to rule out the development of any aneurysm or vascular malformation after revascularization surgery. This could not be seen, but vascular ingrowth through the burr holes and improved capillary perfusion were evident (Fig. 5E).

#### Patient #6

**Fig. 6:**
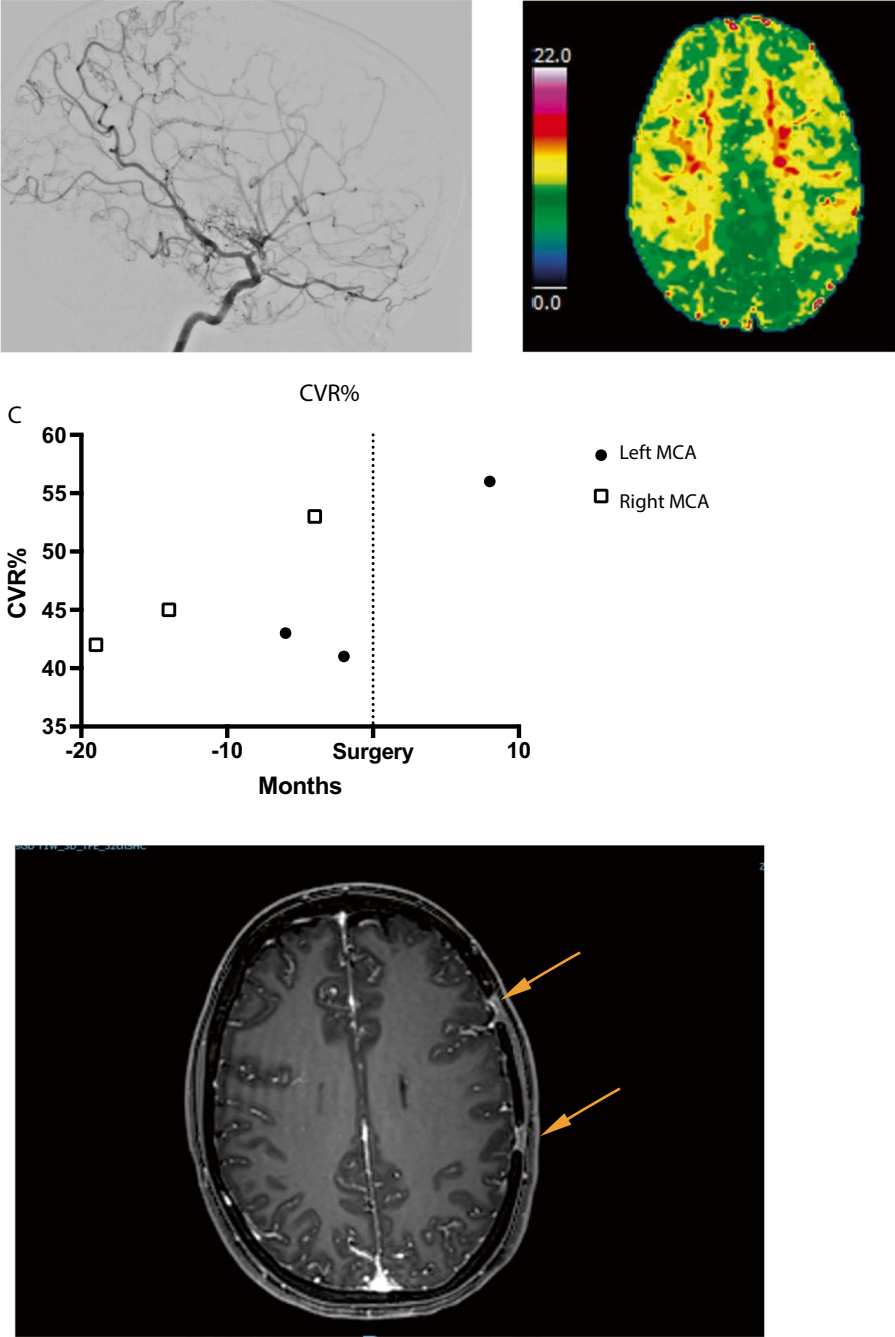
Patient 6, a 26-year-old female with bilateral MMD. **A** DSA of right ICA. **B** TTP map showing delayed TTP bilaterally especially in the MCA territory. **C** CVR on the left side decreased from 43 to 41% pre-op. We started with a left-sided MBH. Post-op MRI showed improved CVR to 56%, and even on the right side, the CVR improved to 53%. Values are presented in relation to when surgery was performed, on each side separately. However, due to symptoms and a strong patient wish, surgery was also performed on the right side. **D** Ingrowth of contrast enhancing blood vessels through burr holes after left-sided MBH

A 26-year-old female presented with headache and transient weakness on her right side. CTA showed bilateral occlusion of the ICA. DSA (Fig. 6) showed bilateral MMD with a Suzuki grade of IV (right) and III (left). ASL-MRI after ACZ challenge showed a CVR of 43% (left) and 42% (right) in the MCA territory bilaterally with no signs of ischemia (Fig. 6C). Due to severe headache and repeated numbness in the right hand and face, a left-sided surgical procedure was carried out 10 months after her initial symptoms. Eight months after the first operation, ASL-MRI with ACZ challenge showed that CVR had increased to 56% on the left side (and to 53% on the right side). A right-sided MBH operation was subsequently performed based upon radiological findings and patient wish. Post-op MRI showed ingrowth of vessels through the burr holes (Fig. 6D).

### Cases managed without surgery

In some cases, surgery was not performed, and patients were monitored based on grading of symptoms, radiological findings and CVR results (Table 4). In general, these cases had better CVRs (ranging from 39 to 84%), no alarming symptoms and no recent ischemic events.

## Discussion

In this study, we used ASL-MRI with the ACZ challenge to assess and monitor patients with MMA before and after revascularization surgery with the MBH technique. We report here our experience in 11 patients.

### ASL-MRI assessment of MMA patients

MMA patients need to be evaluated regarding symptoms, presence of ischemic lesions as well as their capacity of hemodynamic reserve (CVR). This may require repeated radiological examinations, with considerable drawbacks especially in children, where limitation of exposure to radiation is crucial. Many of these disadvantages are overcome with MRI techniques such as ASL [28, 38] and blood oxygen level-dependent (BOLD) MRI [6]. The ASL technique has recently been used for MMA evaluation in several studies in adults [12, 13] and in children [19, 32]. When ASL-MRI was used to study changes in CBF after revascularization surgery in children with MMD [19, 32], increases in CBF correlated with the degree of collateral formation after superficial temporal artery encephaloduroarteriosynangiosis [19]. After direct revascularization, ASL-MRI has also been used to capture early and late changes in CBF [32-34, 42]. Quon et al. observed a general increase in CBF in the MCA territory, but not in all patients [32]. However, there is a lack of studies using ASL-MRI after MBH revascularization to quantify improvement in CVR. There is one study reporting decreased frontal white-matter MRI-ADC diffusion and improved cognitive flexibility after burr hole surgery [4]. In our assessment of MMA patients, the evaluation included measurements of hemodynamic reserve with ASL-MRI and the ACZ challenge in combination with clinical evaluation of symptoms and findings on conventional diagnostic imaging. The outline of our MMA assessment protocol is summarized in Fig. 1. In principle, if symptoms were mild and CVR was stable and relatively high, patients were monitored, and new ASL-MRI examinations with ACZ challenge were performed at approximately 12- to 18-month intervals. If symptoms were severe or CVR was low, or if there were mild symptoms but a steadily decreasing CVR trend on repeated examinations, patients were selected for revascularization surgery with MBH. The CVR was lower in patients selected for surgery (38.5%) compared to the monitored patients (56%). However, the individual response varied; one surgical patient had a pre-op CVR of 60% (Table 4). This patient was the first in our ASL-MR series, and there were some technical issues related to this first examination, and for this reason, he was not included in pre- vs. post-op comparisons. We therefore believe that regarding patient selection for revascularization surgery, we cannot solely rely on one parameter, instead there must be a combined judgment based on symptoms, presence of ischemic lesions, grading of vasculopathy, CVR and patient wish. Regarding cerebral perfusion after MBH surgery, in this small series, we did not perform any statistical comparing between groups due to the low number of patients and heterogeneous nature of the patient population, but in our descriptive statistics, we noticed a relative improvement in CVR after surgery and the ingrowth of vessels through burr holes indicating an improved cerebrovascular status after MBH surgery. Similar neovascular effects were reported by Sainte-Rose et al. 3 months after MBH [35] and also by Oliviera et al. [29]. Post-op CVR varied considerably, e.g., in one patient the CVR decreased somewhat after surgery. Rao et al. also recently reported a negative effect on CVR in 27% of operated cases [33]. Patients with low CVR improvement after surgery could have a greater risk of new ischemic events [33, 36], and we therefore believe these patients should be followed up for a longer time period.

We did not specifically investigate the degree of vessel ingrowth because the ASL-MRI technique was not sensitive enough. However, based on follow-up MRI-TOF (time of flight) sequences, we saw clear signs of vessel ingrowth in all patients. In a previous MBH study, there was ingrowth of vessels in 41/43 burr holes [23] and in 151 of 160 burr holes using a combined approach of dural inversion and periosteal synangiosis [43]. De Oliveira et al. also noticed significant neoangiogenesis through the burr holes [29]. Thus, this fairly simple and safe surgical technique leads to ingrowth of blood vessels from the external circulation to the brain and improved hemodynamic status in MMA. In the future, we need to go more into detail regarding differences between children and adults in the extent of neovascularization after MBH, but this was not within the scope of the present study, instead we wanted to describe our initial experiences using ASL-MRI as an evaluation tool in MMD surgery.

### Overall clinical outcome

After surgery, there was an overall improvement in symptoms, and no new ischemic events were seen in any patient. Also, no other serious events such as CSF leakage or subdural hematoma were seen, despite the usage of perioperative aspirin. We had one serious intracerebral hemorrhagic event in a young girl 3 years after surgery, but she recovered fully after surgical evacuation of the hematoma. This patient was subsequently examined with a post-op DSA that showed no vessel malformation/AV shunting after surgery. Instead, numerous new vessels had been formed through burr holes, and there was improved cerebral capillary perfusion bilaterally. We did not routinely examine all patients with post-op DSA since we did not think this was indicated because the patients had no ischemic symptoms, instead they were monitored with ASL-MRI including the ACZ challenge.

### Methodological considerations

Some methodological considerations regarding the MRI technique need to be discussed. Thus, it is obvious that all the present techniques for quantification of the hemodynamic status of CBF and CVR have their strengths and limitations. In this diverse group of moyamoya patients, the research field is striving to identify the most effective MRI techniques for characterizing cerebral perfusion; however, the most appropriate method has not finally been established. Ultimately, the aim is to adopt a safe and reliable quantitative approach to measure cerebral vascular reserve and cerebral perfusion. An issue regarding the current recommended implementation of ASL, with single delay-based acquisitions, is the inherent dependency on arterial transit time. Arterial transit time artifacts affecting CBF quantification may appear in patients with slow blood flow [39, 41] and are commonly seen in patients with cerebrovascular diseases such as MMS. We have previously demonstrated that overestimation of the effects of arterial transit time artifacts does not significantly affect CBF measurements [9]. However, in the presence of severely delayed arterial blood flow, underestimation of CBF may occur when no or little magnetically labeled blood has reached the imaging volume in time for the readout. The extent of these effects is dependent on the severity of the delay flow and the post-label delay used during the ASL acquisition [11]. To mitigate the underestimation of the CBF, we used a post-label delay of 2500 ms in the present study. However, the drawback is a decreased signal-to-noise ratio due to the T1 relaxation of arterial blood. All ASL datasets included in this study were reviewed visually to exclude vascular territories with a severe delay-induced underestimation of CBF, and no exclusions were made. Still, there may be vascular territories that can be moderately affected.

Recently, multi-delay-based ASL approaches have gained a more widespread application and are recommended in several reports as the first choice over single delay-based ASL in patients with cerebrovascular diseases, if available [20]. Recent studies have shown the potential of CBF and CVR measurements using both single delay- and multi-delay-based ASL for follow-up in MM [11, 12, 19, 32, 33, 42, 44].

Velocity-selective ASL (VSASL) is a novel ASL-based method that is insensitive to delayed arterial blood flow and reduced ASL signal decay due to T1 relaxation [31]. In a recent study, Bolar et al. demonstrated the promising potential of VS-ASL in comparison to pulsed ASL in pediatric patients with MM [3]. However, pulsed ASL is inferior in comparison to pCASL in terms of the signal-to-noise ratio. Moreover, implementation of VSASL has challenges such as sensitivity to eddy currents, electromagnetic field in-homogeneities, diffusion attenuation, patient movement and CSF contamination, the last an issue which becomes more problematic in the aging brain [31]. However, both multi-delay ASL and VSASL have very limited commercial availability, which affects their accessibility.

Another method to estimate CVR is the BOLD MRI technique, exploiting the difference in MRI signal between oxyhemoglobin and deoxyhemoglobin, oxyhemoglobin having higher signal. An increase in blood flow will thus cause increased BOLD signal. Using this technique, the CVR has been assessed with breath-holding or inhaling of carbon dioxide as a vasoactive stimulus [6, 7]. A major drawback with this technique, however, is its qualitative nature that does not permit quantitative measures of blood flow. Another limitation is that the BOLD signal has a multifactorial origin that depends on blood flow as well as other factors, e.g., CMRO_2_ [21].

### Limitations of the study

This study has several limitations related to the low number of included patients and their heterogeneity with regard to age and pathology; therefore, the results have to be interpreted with caution. Further studies including more patients will be needed to corroborate our findings.

## Conclusions

We believe that a combined judgment based on different radiological modalities, such as ASL-MRI with ACZ challenge, together with clinical examinations offers good possibilities to decide the timing of indirect revascularization of MMA patients, thereby limiting revascularization surgery to patients that have a clear need for it.

## Supplementary Information

Below is the link to the electronic supplementary material.

Supplement Fig. 1 (PNG 147 kb)

High resolution image (EPS 1007 kb)

## Data Availability

The datasets generated during and/or analyzed dur ing the current study are available from the corresponding author on reasonable request.
