# Supplementary figures and images for: ASL-MRI-guided evaluation of multiple burr hole revascularization surgery in Moyamoya disease
Source: Acta Neurochir (Wien). 2023 Jun 16;165(8):2057–69. doi: 10.1007/s00701-023-05641-3 (PMC10409847; doi:10.1007/s00701-023-05641-3)

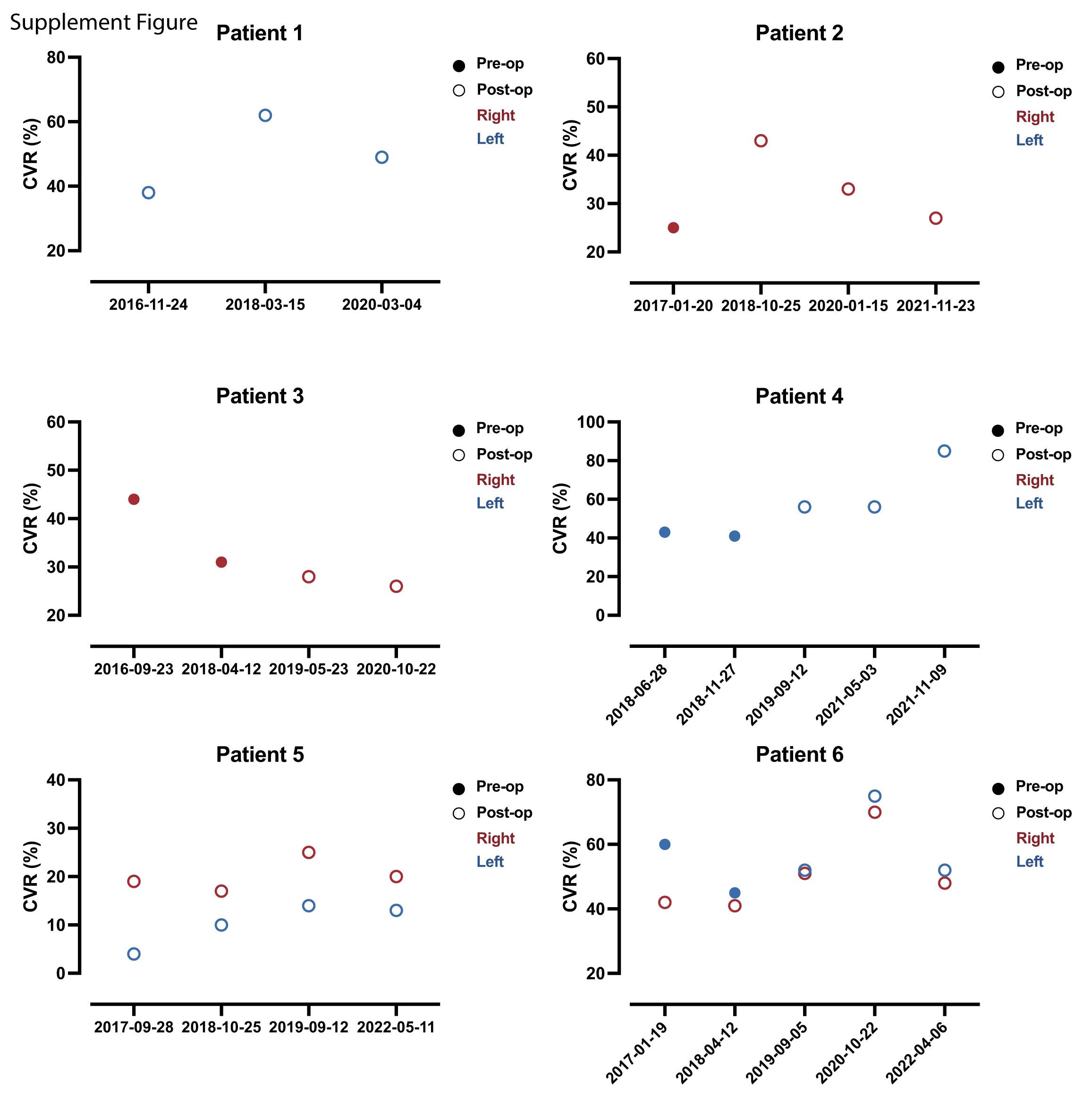

Supplement: Supplementary file 1 — (PNG 147 kb) [file 701_2023_5641_Fig7_ESM.png]
